# Cloning of the Cytochrome *b* Gene From the Tomato Powdery Mildew Fungus *Leveillula taurica* Reveals High Levels of Allelic Variation and Heteroplasmy for the G143A Mutation

**DOI:** 10.3389/fmicb.2019.00663

**Published:** 2019-04-10

**Authors:** Sandra Mosquera, Li-Hung Chen, Brenna Aegerter, Eugene Miyao, Anthony Salvucci, Ti-Cheng Chang, Lynn Epstein, Ioannis Stergiopoulos

**Affiliations:** ^1^Department of Plant Pathology, University of California, Davis, Davis, CA, United States; ^2^University of California Cooperative Extension, Stockton, CA, United States; ^3^University of California Cooperative Extension, Woodland, CA, United States

**Keywords:** cytochrome b, fungicide resistance, heteroplasmy, mitochondria, mutations, polymorphisms, Q_o_I fungicides, strobilurin

## Abstract

*Leveillula taurica* is a major pathogen of tomato and several other crops that can cause substantial yield losses in favorable conditions for the fungus. Quinone outside inhibitor fungicides (Q_o_Is) are routinely used for the control of the pathogen in tomato fields across California, but their recurrent use could lead to the emergence of resistance against these compounds. Here, we partially cloned the cytochrome *b* gene from *L. taurica* (*Lt cytb*) and searched within populations of the fungus collected from tomato fields across California for mutations that confer resistance to Q_o_Is. A total of 21 single nucleotide polymorphisms (SNPs) were identified within a 704 bp fragment of the *Lt cytb* gene analyzed, of which five were non-synonymous substitutions. Among the most frequent SNPs encountered within field populations of the pathogen was the G143A substitution that confers high levels of resistance against Q_o_Is in several fungi. The other four amino acid substitutions were novel mutations, whose effect on Q_o_I resistance is currently unknown. Sequencing of the *Lt cytb* gene from individual single-cell conidia of the fungus further revealed that most SNPs, including the one leading to the G143A substitution, were present in a heteroplasmic state, indicating the co-existence of multiple mitotypes in single cells. Analysis of the field samples showed that the G143A substitution is predominantly heteroplasmic also within field populations of *L. taurica* in California, suggesting that Q_o_I resistance in this fungus is likely to be quantitative rather than qualitative.

## Introduction

Powdery mildews are obligate biotrophic fungi that cause extensive damage in a wide range of crops (Ridout, 2009). In California, powdery mildew infections on tomato are caused by the fungal species *Leveillula taurica* (Correll et al., 1987) and *Oidium neolycopersici* (Jones et al., 2001), which are primarily associated with the disease in open field and greenhouse cultivated tomatoes, respectively (Correll et al., 1987; Cerkauskas and Brown, 2015). Unlike most powdery mildew fungi that are host specific, these two species have a wide host range within the Solanaceae, Alliaceae, and Cucurbitaceae plant families, thus making them extremely destructive pathogens in a number of vegetable crops (Correll et al., 1987; Jones et al., 2001). A third species, *Oidium lycopersici*, is also known to infect tomato but it is primarily reported only in Australia (Kiss et al., 2001) and, during the last few years, it has also occurred sporadically in California as well (Salvucci et al., 2016). In the past, *O. neolycopersici* and *O. lycopersici* were mistaken for a single species, but molecular data has clearly shown that they are distinct (Kiss et al., 2001).

**FIGURE 1:**
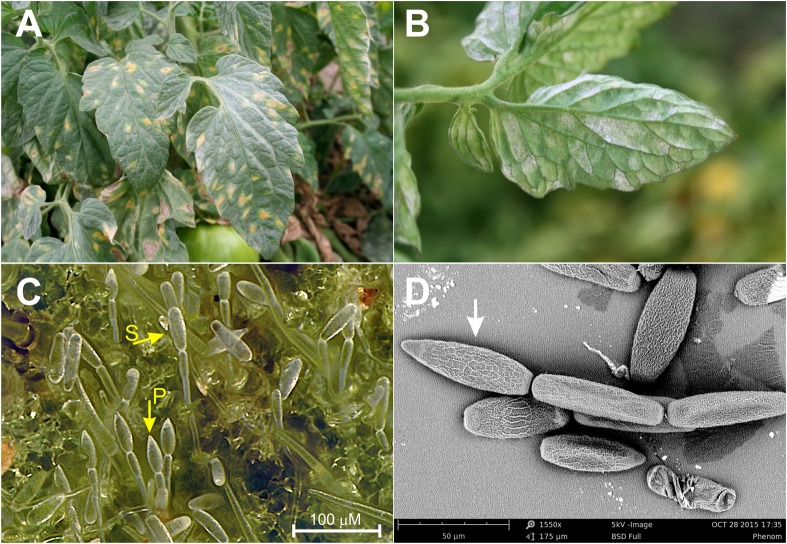
Disease symptoms caused by *Leveillula taurica* on tomatoes. **(A)** Disease symptoms as mostly seen on the adaxial leaf side. **(B)** Disease as mostly seen on the abaxial leaf side. **(C)** Primary (P) and secondary (S) conidia of *L. taurica* produced on infected tomato leaf tissue (magnification ca. 960×). **(D)** Scanning electron microscopy image of the apical lanceolate-shaped conidium (pointed by an arrow) produced by *L. taurica* (magnification 1,550×).

Although all three species are known to infect tomatoes in California, powdery mildew epidemics in the fields are predominately caused by *L. taurica* (Correll et al., 1987; Aegerter et al., 2014). The pathogen was first reported in California in 1978 and has since become a recurrent problem in nearly all tomato-growing districts of the state (Correll et al., 1987; Aegerter et al., 2014). Warm, arid and semiarid climatic conditions seem to promote infections by *L. taurica*, which under favorable conditions can have a significant impact on fruit production and quality, reducing yields by up to 40% if left uncontrolled (Jones and Thomson, 1987; Aegerter et al., 2014). The fungus mainly infects the leaves, where it enters through the stomata and grows predominately endophytically, penetrating deeply into the mesophyll of the leaf tissue (Thomson and Jones, 1981; Palti, 1988). This is a fundamental difference between *L. taurica* and the other powdery mildew species infecting tomato that are primarily epiphytic (Jones et al., 2001; Kiss et al., 2001). Reproductive spores produced by the three species are also markedly different, with *L. taurica* producing large, single-celled, lanceolate primary conidia and secondary cylindrical conidia in chains that emerge from the abaxial leaf side (Thomson and Jones, 1981; Correll et al., 1987; Palti, 1988) (Figure 1). In contrast, *O. neolycopersici* and *O. lycopersici* produce conidia singly or in pseudochains of 2–6 ellipsoid to cylindrical shaped conidia on the adaxial side (Jones et al., 2001). Macroscopically, infections by *L. taurica* are primarily seen as white patches of asexual spores on the abaxial leaf side that appear as irregular in shape chlorotic to yellowish spots on the adaxial side (Figure 1). Infected leaves, eventually turn yellow to necrotic and become shriveled, whereas defoliation can occur in severe infections (Correll et al., 1987).

California produces over 96% of processing tomatoes in the United States, and consequently effective management of *L. taurica* is crucial to the economic vitality of the tomato industry and United States agriculture. With only a few mildew-resistant varieties available, current control methods rely mostly on the use of sulfur-dust and commercial fungicides such as demethylation inhibitors (DMIs) and quinone outside inhibitors (Q_o_Is, also known as strobilurins) (Guzman-Plazola, 1997). However, the continuous use of these fungicides increases the danger for fungicide resistance development and cases of disease control failure due to resistance development against these two fungicides classes have already been reported in several plant pathogens (Ma and Michailides, 2005).

Although multiple mechanisms may confer resistance against fungicides in fungi, it is most frequently triggered by point mutations in the fungicides’ target sites such as the sterol 14α-demethylase (*CYP51*) and cytochrome b (*cytb*) genes, which encode for the enzymatic targets of DMI and Q_o_I fungicides, respectively (Bartlett et al., 2002; Lupetti et al., 2002). The mode of action of Q_o_Is is based on blocking mitochondrial respiration and thus energy production in the cells, by binding to the cytochrome bc1 enzyme complex (complex III) at the Q_o_ site that is partially encoded by the mitochondrial *cytb* gene (Bartlett et al., 2002; Gisi et al., 2002; Fisher and Meunier, 2008). Due to their site-specific mode of action, the risk of resistance development against Q_o_Is is considered high and, to date, Q_o_I resistance is reported in many plant pathogenic fungi around the world, including powdery mildews (Ishii et al., 2001; Gisi et al., 2002; Baudoin et al., 2008; Fisher and Meunier, 2008; Fernández-Ortuño et al., 2010; Vielba-Fernandez et al., 2018). In most cases, target-site mutations are the primary molecular mechanism associated with Q_o_I resistance and at least 21 amino acid substitutions have been described in filamentous fungi, yeasts, and protozoan parasites as conferring resistance to these compounds (Gisi et al., 2002; Fisher and Meunier, 2008). Most of the mutations are concentrated at the Q_o_ site of cytb and cause changes in the structure of the enzyme’s active site that prevent binding of the Q_o_Is to it (Fisher and Meunier, 2008). Three amino acid substitutions are predominately observed in field populations of Q_o_I-resistant fungal pathogens of agronomic importance, i.e., an exchange from Phe-to-Leu at position 129 (F129L), an exchange from Gly-to-Arg at position 137 (G137R), and an exchange from Gly-to-Ala at position 143 (G143A) (Gisi et al., 2002; Fernández-Ortuño et al., 2010). Although all three mutations can lead to the development of Q_o_I resistance in the fields, resistance factors associated with them may differ, as strains carrying the G143A substitution frequently exhibit higher levels of resistance to Q_o_Is compared to strains that carry the G137R or F129L mutations (Gisi et al., 2002; Fisher and Meunier, 2008; Fernández-Ortuño et al., 2010). However, the G143A substitution may also impede the activity of cytb in some fungi, thus resulting in a fitness penalty and poor competitiveness against wild-type strains. If this is the case, then the dynamics of Q_o_I resistant and sensitive strains in fungal populations may shift depending on the intensity of the selection pressure imposed by the fungicide (Gisi et al., 2002; Fisher and Meunier, 2008; Fernández-Ortuño et al., 2010).

To moderate the fitness cost associated with the G143A mutation, many fungi are shown to exhibit mitochondrial heteroplasmy for the *cytb* gene and particularly for the G143A substitution (Lesemann et al., 2006; Villani and Cox, 2014; Vielba-Fernandez et al., 2018). Heteroplasmy is commonly described as the coexistence of more than one type of mitochondrial genomes (mitotypes) in a single cell and although it regularly occurs in plants, it is less often described in fungi (Barr et al., 2005; Woloszynska, 2009; Wilson and Xu, 2012). As there can be many mitochondria in a cell, mitochondrial heteroplasmy for the G143A mutation means that some mitochondria carry the mutated A143 allele that confers resistance to Q_o_Is while others have the wild-type G143 allele that compensates for the dysfunctional one. If this is the case, then the balance between fitness cost, on the one hand, and the levels of resistance against Q_o_Is, on the other, in different strains of a fungus can be determined by the ratio between mutant versus wild-type mitochondria (Lesemann et al., 2006). Notably, the ratio between wild-type and mutant cytb alleles in heteroplasmic fungal cells is not fixed but can change depending on the selection pressure imposed by the application frequency of Q_o_I fungicides (Ishii et al., 2001). Therefore, information on the presence or absence of mitochondrial heteroplasmy with respect to mutations in cytb that lead to resistance against Q_o_Is, can be of practical relevance when designing resistance management programs and fungicide rotation schemes.

The aim of this study was to clone the *cytb* gene from *L. taurica* and examine whether strains with mutations that are known to confer resistance to Q_o_Is, such as the F129L, G137R, and G143A substitutions, are already present within populations of the fungus infecting tomatoes in open field cultivation in California. Such information is critical to improving disease control and for developing fungicide resistance management programs for this pathogen.

## Materials and Methods

### Cloning of the *L. taurica cytb* Gene (*Lt cytb*)

To clone the *cytb* gene from *L. taurica* (*Lt cytb*), a PCR-based approach was followed using primers RSCBF1 and RSCBR2 that have been successfully used in the past for the amplification of the *cytb* gene from various fungal species, including powdery mildews (Supplementary Table S1) (Ishii et al., 2001; Lesemann et al., 2006; Zhang et al., 2009). Spores of *L. taurica* were collected from heavily infected tomato leaves and used for DNA extraction. The presence of *L. taurica* and the concurrent absence of *O. lycopersici* or *O. neolycopersici* in the DNA sample was confirmed by PCR, using gene-specific primers that amplified the internal transcribed spacer (ITS) regions of each species (Supplementary Table S1). These primers were designed from the rDNA sequences of the three fungal species available in NCBI and their specificity was first examined in PCR reactions that used DNA from the other two species as a template (Supplementary Figure S1).

PCRs targeting the amplification of the ITS region were performed using the iProofTM High-Fidelity PCR Kit (BIO-RAD, Hercules, CA, United States) according to the manufacturer’s reaction and cycling protocol, using an annealing temperature of 59°C. PCR reactions with the RSCBF1/RSCBR2 primer pair targeting the amplification of the *Lt cytb* gene were performed using the Phusion High-Fidelity DNA Polymerase (New England Biolabs, Ipswich, MA, United States) according to the manufacturer’s recommended reaction and cycling protocol, using an annealing temperature of 58°C. Following the PCR reactions, products were cloned into pGEMT-Easy vector (Promega, Madison, WI, United States) according to the manufacturer’s instructions and sequenced at Quintara Bio (San Francisco, CA, United States). Sequences were analyzed by BLAST searching (Altschul et al., 1997) them in the nr database of NCBI and clones showing similarity to *cytb* genes from other fungi were considered as positive hits for the *Lt cytb*.

To obtain additional sequences at the 5′- and 3′-end of the *Lt cytb*, the gene fragment that was cloned using the RSCBF1/RSCBR2 primers was blasted against the NCBI and BLASTn hits with higher than 85% identity at the nucleotide level to the *Lt cytb* fragment were aligned with Clustal Omega (Sievers et al., 2011). The produced alignment then served as a basis for designing a set of three forward and reverse degenerate primers (Supplementary Table S1) that along with the RSCBF1 and RSCBR2 primers were used in various combinations to amplify additional *cytb* gene sequences from *L. taurica*. One of these primer combinations, i.e., RSCBF1/Cytb-MildDeg-R3, generated an additional 551 bp downstream of the 286 bp *Lt cytb* fragment obtained using the RSCBF1/RSCBR2 primer pair (Supplementary Figure S2). However, all other primer combinations failed to yield additional sequences upstream or downstream of the amplified *cytb* gene fragment. Therefore, a DNA walking approach was also followed using the Universal GenomeWalker 2.0 kit (Clontech Laboratories, Mountain View, CA, United States) according to the manufacturer’s instruction. Briefly, genomic DNA of *L. taurica* was digested with the *Stu*I restriction enzyme and ligated to the genome walker adaptor provided by the kit. Two gene-specific primers, i.e., Lt_cytb_5RACE_R1 and Lt_cytb_3RACE_F1 were used in combination with the adaptor primer AP1 (Supplementary Table S1) for the primary PCR, whereas two additional gene-specific primers, i.e., Lt_cytb_5end_R3 and Lt_cytb_3RACE_F2 were used in combination with the adaptor primer AP2 (Supplementary Table S1) for the secondary nested PCR. All four primers were designed on the partial *Lt cytb* sequence described above. Primer pairs Lt_cytb_5end_R3/AP2 successfully amplified a DNA fragment of ∼400 bp in size, and Lt_cytb_3RACE_F2/AP2 amplified a DNA fragment of ∼750 bp in size. Unfortunately, sequencing of these two DNA fragments revealed that they had no homology to *cytb* sequences and thus they were likely non-specific products.

Although the complete *Lt cytb* gene could not be obtained, the sequence of the 837 bp long *cytb* fragment that was successfully cloned from *L. taurica* has been deposited in NCBI under the Acc. no. MK015626.

### Collection of *L. taurica* Infected Leaves From Tomato Fields and Identification of Mutations Within the Cloned *Lt cytb* Fragment

Tomato leaves with clear symptoms of infections by *L. taurica* (e.g., sporulation of the fungus on the abaxial leaf side with the distinctive lanceolate conidia at the tip of conidial chains in the sporulating areas) (Figure 1) were periodically collected from July through October 2015, from 22 fields distributed in five counties in California, i.e., Contra Costa (1 field), Fresno (3), San Joaquin (7), Solano (3), and Yolo (9). Sampling was done by collecting multiple compound leaves from a number of plants that were randomly distributed within each field. Information about the samples, such as location, collection date, tomato variety (if known), and fungicide-spray history of the tomato field (if shared by the farmer), was gathered and recorded (Supplementary Table S2). In the lab, 1.5 cm diameter disks of infected leaf tissues were excised from individual leaflets using a flame-sterilized cork-borer, placed individually in a 2.0 mL microcentrifuge tube, and stored at -80°C until DNA extraction. These were performed using the DNeasy Plant Mini Kit protocol (QIAGEN, Germantown, MD, United States) according to the manufacturer’s instructions. DNA was eluted in 100 μL of sterile MQ water and was further diluted 10–30× before being used as template in subsequent PCR reactions for the amplification of the *cytb* gene. The presence of *L. taurica* and the concurrent absence of *O. lycopersici* and *O. neolycopersici* in the samples was confirmed by PCR, as described above. Amplification of the *Lt cytb* gene was performed using primers Lt-RSCBF1, a modified RSCBF1 primer to match the specific *L. taurica* 5′-end sequence, and Lt-Cytb.GSP-R2, which is nested to the degenerate Cytb-MildDeg-R3 primer (Supplementary Table S1 and Supplementary Figure S2). The specificity of this primer pair was examined in PCR reactions using DNA of *L. taurica, O. lycopersici*, or *O. neolycopersici* as template, where it was confirmed that it only amplifies the *cytb* gene from *L. taurica* and not from the other two species (Supplementary Figure S1). PCR reactions using the Lt-RSCBF1/Lt-Cytb.GSP-R2 primer pair were performed using the Phusion High-Fidelity DNA Polymerase (New England Biolabs, Ipswich, MA, United States) according to the manufacturer’s recommended reaction and cycling conditions, with an annealing temperature of 59°C. For all samples analyzed, the 798 bp amplification product obtained from the PCR reaction was loaded in a 1.2% agarose gel and was gel excised using the Zymoclean Gel DNA Recovery Kit (Zymo Research, Irvine, CA, United States) according to the manufacturer’s protocol. Sequencing was done on the positive strand using primer Lt-RSCBF1. Sequences were aligned using the MEGA software package (Version 6.0) (Tamura et al., 2013) and electropherograms were manually examined for the presence of overlapping electrophoretic peaks at polymorphic positions. Polymorphic data were analyzed using DNAsp (Version 5) (Librado and Rozas, 2009). The peak intensities at positions with mixed bases, were quantified using the Ab1 Peak Reporter software tool (Roy and Schreiber, 2014) from Life Technologies (Life Technologies, Thermo Fisher Scientific, Carlsbad, CA, United States). The major and minor peaks corresponding to polymorphic nucleotides were determined by a sliding window analysis using an in-house Perl script. The peak height ratio was calculated by dividing the intensity of the minor peak by the major peak.

### Amplification of the *cytb* Gene From Individual Single-Cell Conidia of *L. taurica*

Conidia of *L. taurica* were collected from infected tomato leaves by gently discharging the spores onto a microscope slide (Fisher Scientific). Each conidium was then transferred using a paintbrush hair and tweezers into separate 200 μL PCR tubes containing 5 μL of nucleases/nucleic acids-free PCR grade water (Fisher Scientific, Hampton, NH, United States), and the tubes were incubated for 20 min at 100°C. The heated spore suspensions were then used as template to amplify the partial *Lt cytb* gene with primers Lt-RCBF1 and Lev-Cytb-GSP-R2 (Supplementary Table S1), as described above. PCR products were visualized on a 1.2% agarose gel and gel-purified with the Zymoclean Gel DNA Recovery Kit (Zymo Research, Irvine, CA, United States) according to the manufacturer’s protocol. To obtain sufficient product for subsequent sequencing, the 798 bp *Lt cytb* fragment amplified from the individual conidia was re-amplified using OneTaq DNA Polymerase (New England Biolabs) according to the manufacturer’s specifications, using 1 μL of the purified PCR fragment as template and the same primers used in the first PCR reaction for amplification. The PCR product was again gel purified as described above and sequenced using the Lt-RCBF1 primer. Sequences were analyzed as described above.

## Results

### Molecular Characterization of the *Lt cytb* Gene

Amplification using DNA of *L. taurica* as a template with primers RSCBF1 and RSCBR2 (Ishii et al., 2001) generated a 286 bp fragment (Supplementary Figure S1) that based on BLASTn analysis showed highest similarity to the *cytb* genes from the powdery mildew fungi *Podosphaera leucotricha* (99% identity, 75% coverage) and *Podosphaera fusca* (90% identity, 96% coverage). When translated with the yeast mitochondrial genetic code, the 95 amino acid long product spanned the C-terminal region of transmembrane helix C and the surface cd1 loop region of cytb that contain three of the residues associated with resistance to Q_o_Is in fungal plant pathogens, i.e., F129, G137, and G143 (Gisi et al., 2002; Fisher and Meunier, 2008). Subsequent PCRs using the primer combination RSCBF1/Cytb-MildDeg-R3 (Supplementary Table S1) generated an additional 551 bp downstream of the 286 bp fragment, which increased the length of the cloned *Lt cytb* gene fragment to 837 bp (Supplementary Figure S1). Unfortunately, despite repeated attempts, all efforts to obtain sequences upstream of this fragment were unsuccessful.

A BLASTn search against the NCBI nr database showed that the cloned 837 bp *Lt cytb* gene fragment exhibits high levels of nucleotide identity to the *cytb* gene sequences from the powdery mildew species *Erysiphe polygoni* (99% identity, 68% coverage) and *P. fusca* (98% identity, 67% coverage) and somewhat lower levels of identity to *cytb* genes from the non-powdery mildew species *Zasmidium cellare* (86% identity, 100% coverage), *Phialocephala subalpina* (86% identity, 100% coverage), *Rhynchosporium orthosporum* (86% identity, 100% coverage), and other Ascomycete fungi (Table 1). When translated with the yeast mitochondrial genetic code, the 837 bp *Lt cytb* gene fragment produces a 278 amino acid long peptide product with no in-frame stop codons (Supplementary Figure S1), suggesting the absence of any introns within the nucleotide sequence. BLASTp analysis showed that the 278 amino acid peptide exhibits high similarity to the cytb of *Cercospora beticola* (86% identity, 96% similarity), *Pseudocercospora mori* (86% identity, 96% similarity), *Graphis lineola* (85% identity, 96% similarity), *Botryosphaeria dothidea* (86% identity, 96% similarity), *Z. cellare* (86% identity, 96% similarity), and others (Table 1). The cytb peptide sequences of *E. polygoni* and *P. fusca* were not included within the top 100 significant BLASTp hits obtained using the *Lt* cytb peptide as query, possibly because only partial sequences of the corresponding coding genes have been deposited in NCBI. Nonetheless, alignment using blast2seq (BLAST 2 sequences) of the partial *Lt* cytb peptide sequence with that of *E. polygoni* (AHL44336.1) showed that the two share 91% identity and 97% similarity over 192 aligned amino acids, whereas alignment with the partial cytb peptide sequence from *P. fusca* (ABL75948.1) showed that they share 90% identity and 96% similarity over 190 aligned amino acids (Table 1). Thus, as expected, the characterized *Lt* cytb peptide shows significantly higher levels of identity both at the nucleotide and protein level to cytb protein sequences from other powdery mildew species within the *Erysiphaceae* family, class of *Erysiphales*.

**Table 1:** Description of the top five BLASTn and BLASTp hits retrieved from the NCBI nr database, using as queries the 837 bp cytochrome *b* fragment cloned from *Leveillula taurica* (*Lt cytb*) and the translated with the yeast mitochondrial genetic code 278 amino acid (aa) peptide, respectively.

Description	Accession number	Hit length	Hit coverage	Query coverage	*E*-value	% Identity	% Similarity
			
BLASTn hits^1^		bp	bp	bp			
*Erysiphe polygoni* isolate UTC206 cytochrome *b (cytb)* gene, partial cds; mitochondrial	KF925325.1	575	1–575	86–660	0.0	99	na
*Podosphaera fusca* isolate 23785 cytochrome *b (cytb)* gene, partial cds; mitochondrial	EF137834.1	569	1–561	110–670	0.0	98	na
*Zasmidium cellare* mitochondrion, complete genome	NC_030334.1	1164	201–1034	1–834	0.0	86	na
*Phialocephala subalpina* voucher UAMH:11012 mitochondrion, complete genome	JN031566.1	1167	201–1037	1–837	0.0	86	na
*Rhynchosporium orthosporum* mitochondrion, complete genome	KF650574.1	1161	201–1037	1–837	0.0	86	na

**BLASTp hits^1^**		**aa**	**aa**	**aa**			

Cytochrome b, partial (mitochondrion) *Cercospora beticola*	ABM53468.1	321	4–280	1–278	2e^-180^	86	96
Cytochrome b (mitochondrion) *Pseudocercospora mori*	YP_009469553.1	390	68–345	1–278	2e^-180^	86	96
Cytochrome b, partial (mitochondrion) *Graphis lineola*	YP_009424478.1	394	68–345	1–278	1e^-179^	85	96
Apocytochrome b (mitochondrion) *Botryosphaeria dothidea*	AWN55983.1	385	68–345	1–278	3e^-179^	86	96
Cytochrome b (mitochondrion) *Zasmidium cellare*	YP_009257391.1	387	68–345	1–278	2e^-178^	85	96

**Blast2seq^**2**^**							

Cytochrome b, partial (mitochondrion) *Erysiphe polygoni*	AHL44336.1	192	1–192	29–220	4e^-134^	91	97
Cytochrome b, partial (mitochondrion) *Podosphaera fusca*	ABL75948.1	190	1–190	37–226	1e^-130^	90	96

*^1^Significant hits from multiple strains of the same species are not listed. ^2^The BLASTp reports obtained from pairwise alignments using Blast2seq of the 278 amino acid Lt cytb sequence with the partial cytb sequences of Erysiphe polygoni and Podosphaera fusca are also indicated.*

**FIGURE 2:**
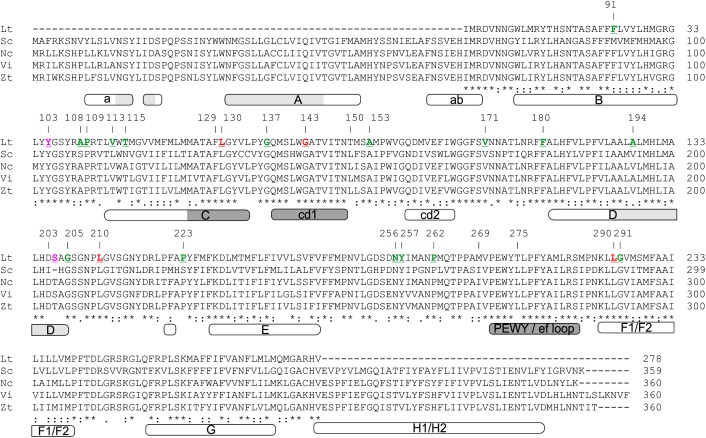
Amino acid alignment of the partial cytochrome b peptide sequence from *L. taurica* (*Lt* cytb) with well characterized cytb protein sequences from representative Ascomycetes, including *Saccharomyces cerevisiae* (Sc: NP_009315.1), *Neurospora crassa* (Nc; Q35128.3), *Venturia inaequalis* (Vi; AAB95255.1), and *Zymoseptoria tritici* (Zt; Q6X9S4.1). The numbering on the top of the alignment is in reference to the *Sc* cytb amino acid sequence. Note that at position 203 the *Sc* cytb is missing one amino acid compared to the cytb sequences from other fungi, and thus hereafter numbering in these fungi is shifted by one position. Polymorphic amino acids in the *Lt* cytb are highlighted in red, whereas amino acids that correspond to silent substitutions in the nucleotide data are shown in green. Magenta colored amino acids correspond to the ones which were detected as polymorphic only in cytb sequences obtained from single conidia of *L. taurica* but not from infected leaf samples from the field. The domain organization of cytb is also shown beneath the sequence alignment. Domain annotations are according to Fisher and Meunier (2008) and numbering is based on the *Sc* cytb amino acid sequence. Domains A, B, C, D, E, F1/F2, G, and H1/H2 represent the transmembrane helices, a and ab are surface domains, whereas cd1, cd2 and PEWY/ef loop are surface exposed loops of the protein that play important role in structuring the Q_o_ binding pocket. The quinol oxidation (Q_o_) and the quinol reduction (Q_i_) sites of the *Sc* cytb are shown in dark and light gray, respectively.

The atomic structure and organization of the primary catalytic sites of cytb such as the sites of quinol reduction (Q_i_) and quinol oxidation (Q_o_) have been well characterized in yeast and other eukaryotic species (Iwata et al., 1998; Zhang et al., 1998; Hunte et al., 2000; Fisher et al., 2004; Fisher and Meunier, 2008). Alignment of the partial *Lt* cytb peptide sequence with the well-characterized full-length cytb proteins from *Saccharomyces cerevisiae* (*Sc* cytb: NP_009315.1) (Foury et al., 1998), *Venturia inaequalis* (*Vi* cytb; AAB95255.1) (Zheng and Köller, 1997), *Zymoseptoria tritici* (*Zt* cytb; Q6X9S4.1) (Fraaije et al., 2005), and *Neurospora crassa* (*Nc* cytb; Q35128.3) (Helmer-Citterich et al., 1983), showed that it spans positions 68–319 of the 385 amino acid long *Sc* cytb protein, or positions 68–320 of the cytb protein sequences from *Vi*, *Zt*, and *Nc* (Figure 2) (Fisher et al., 2004). With reference to the secondary structure of the *Sc* cytb protein sequence, the characterized *Lt* cytb peptide includes the transmembrane helices B, C, D, F1-F2, and G2, the surface cd1 and cd2 helices present between the transmembrane helices C and D, and D and F1, respectively, as well as the ef loop that connects the transmembrane helix F with the cd2 one (Iwata et al., 1998; Fisher and Meunier, 2008). Thus, the primary sequence of the characterized *Lt* cytb peptide includes the entire Q_o_ pocket, which is formed by the surface-exposed cd1 helix (residues 138–149), the PEWY/ef loop (residues 271–284), and the C-terminal region of transmembrane helix C (residues 125–134) (Figure 2) (Iwata et al., 1998; Hunte et al., 2000; Fisher and Meunier, 2008). In contrast, the Q_i_ site, which is formed by the surface helix a and the N-, C-, and N-termini of transmembrane helices A, D, and E, respectively, is only partially represented within the characterized *Lt* cytb peptide. Highly conserved sites in fungal cytb proteins, such as the PEWY motif that forms part of the Q_o_ site, residues H82 and H183, and residues H96 and H197 that are iron ligands within the binding pocket of the *heme b*_L_ and *heme b*_H_ n, respectively (Fisher and Meunier, 2008), are all also conserved in the *Lt* cytb peptide sequence (Figure 2).

### Identification of Polymorphisms Within the Characterized *Lt cytb* Gene Fragment in Field Populations of the Fungus Indicates High Rates of Nucleotide Substitutions

The frequent use of Q_o_Is against *L. taurica* in tomato fields in California increases the risk for resistance development to these compounds. To screen for mutations associated with increased tolerance to Q_o_Is in fungi, such as the F129L, G137R, and G143A substitutions (Gisi et al., 2002), and to identify additional allelic variation within the cloned *Lt cytb* gene fragment, we used the primers Lt-RSCBF1 and Lt-Cytb.GSP-R2 to PCR amplify and subsequently sequence a 798 bp fragment of the *Lt cytb* gene from 104 infected leaf samples. However, the first 58 bp and last 36 bp were excluded from the sequencing analysis due to loss of resolution in the produced sequencing electropherograms. The translated 234 amino acid product of the remaining 704 bp spanned residues 87–319 of the *Sc* cytb or residues 87–320 of the cytb of other Ascomycete fungi (Figure 2) (Fisher et al., 2004).

Twenty-one single nucleotide polymorphisms (SNPs) distributed over 20 codons were identified within the 704 bp *Lt cytb* gene fragment analyzed, indicating a high mutation rate (Table 2). Thirteen of the SNPs were transitions and 10 were transversions, with three being present in the 1st codon position, one in the 2nd, and 17 in the 3rd (Supplementary Figure S2). Eighteen of the nucleotide substitutions were present in multiple samples and three were singletons that were distributed in two samples (Table 2 and Supplementary Table S2). None of the singletons, however, induced an amino acid exchange in the translated peptide. Thirteen of the 18 SNPs that were found in multiple samples were silent substitutions, whereas five were non-synonymous substitutions that resulted in an amino acid exchange in the peptide product. These were a Leu-to-Thr substitution at codon 63 (L63T or L130T with reference to the *Sc* cytb; hereafter L130T), a Gly-to-Ala substitution at codon 76 (G76A or G143A with reference to the *Sc* cytb; hereafter G143A), a Thr-to-Leu substitution at codon 144 (T144L or T210L with reference to the *Sc* cytb; hereafter T210L), and a Thr-to-Leu or Thr-to-Phe substitution at codon 244 (T244L/F or T290L/F with reference to the *Sc* cytb; hereafter T290L/F) (Table 2 and Figure 2). From these, the G143A substitution that has been associated with resistance to Q_o_Is in fungi was frequently present (50/104; 48%) within the *L. taurica* infected leaf samples collected from the field (Supplementary Table S2). However, no substitutions were observed at residues F129L and G137R with reference to the *Sc* cytb, (F62L and G70R in the characterized *Lt* cytb peptide, respectively) that also confer tolerance to Q_o_Is (Gisi et al., 2002) (Figure 2).

**Table 2:** Nucleotide and induced amino acid substitutions detected in the partial cytochrome *b* fragment cloned from *Leveillula taurica*.

Single nucleotide substitutions (SNPs)^1^	Induced amino acid (aa) substitution^2^	Matching aa position in yeast cytb	Single conidia in which SNPs were heteroplasmic	Predicted position in the secondary structure of the yeast cytb
m.73C > T	p.Phe24Phe	p.Phe91	None	TM helix B
m.124G > A	p.Ala41Ala	p.Ser108	None	Loop connecting TM helices B and C
m.127A > T	p.Pro42Pro	p.Pro109	SS2, SS6	Loop connecting TM helices B and C
m.139A > T	p.Val46Val	p.Leu113	None	TM helix C
m.145T > A	p.Thr48Thr	p.Asn115	SS2, SS6	TM helix C
m.188T > C	**p.Leu63Thr**	p.Leu130	SS2, SS6	TM helix C/Q_o_ binding site
m.211G > A	p.Gly70Gly	p.Gly137	SS2, SS6	Loop connecting TM helix C with helix cd1/Q_o_ binding site
m.228G > C	**p.Gly76Ala**	p.Gly143	SS2, SS3, SS4, SS5, SS6, SS8, SS9	Helix cd1/Q_o_ binding site; Induces Q_o_I resistance
m.259T > A	p.Ala86Ala	p.Ala153	SS2, SS6	Loop connecting helix cd1 and cd2
m.313A > T	p.Val104Val	p.Val171	SS2	Loop connecting helix cd2 with TM helix D
m.340C > T	p.Phe113Phe	p.Phe180	SS2, SS6	TM helix D
m.382A > C	pAla127Ala	p.Val194	SS2, SS6	TM helix D/Q_i_ binding site
m.418A > G^3^	p.Gly139Gly	p.Gly205	None	TM helix D/Q_i_ binding site
m.431T > C	**p.Leu144Thr**	p.Leu210	SS2	Loop connecting TM helices D and E
m.472A > T^3^	p.Pro157Pro	p.Ser223	None	Loop connecting TM helices D and E
m.571T > C	p.Asn190Asn	p.Asn256	SS4	Loop connecting TM helices D and F1
m.574T > C^3^	p.Tyr191Tyr	p.Tyr257	SS2	Loop connecting TM helices D and F1
m.589A > T	p.Pro196Pro	p.Pro262	SS4, SS9	Loop connecting TM helices D and F1
m.671T > C	**p.Leu224Thr**	p.Leu290	SS2, SS4, SS6	TM helix F1
m.673A > T	**p.Leu224Phe**	p.Leu290	SS2, SS4	TM helix F1
m.675A > T	p.Gly225Gly	p.Gly291	None	TM helix F1
m.109C > T^4^	p.Tyr36Tyr	p.Tyr103	SS3, SS8	TM helix B
m.410A > G^4^	**p.Ser137Gly**	Gap between p.ile203/His204	SS5, SS8	TM helix D/Q_i_ binding site

*Polymorphisms at the DNA and protein level are described based on the nomenclature proposed by the Human Genome Variation Society (den Dunnen et al., 2016). ^1^Nucleotide numbering is based on the 837 bp cytochrome b fragment cloned from L. taurica with the first nucleotide numbered as +1. Note that base pairs 1–58 and 801–837 are not included in the analysis. ^2^Protein numbering is based on the translated 278 amino acid product of the 837 bp Lt cytb gene fragment with the first residue numbered as +1. Note that the first 19 and last 12 residues are not included in the analysis. ^3^Singleton mutation. ^4^Polymorphisms that were detected only in cytb sequences obtained from single conidia of L. taurica. Bolded terms correspond to non-synonymous amino acid substitutions in the Lt cytb.*

### The *Lt cytb* Gene Is Highly Heteroplasmic at Polymorphic Sites

**FIGURE 3:**
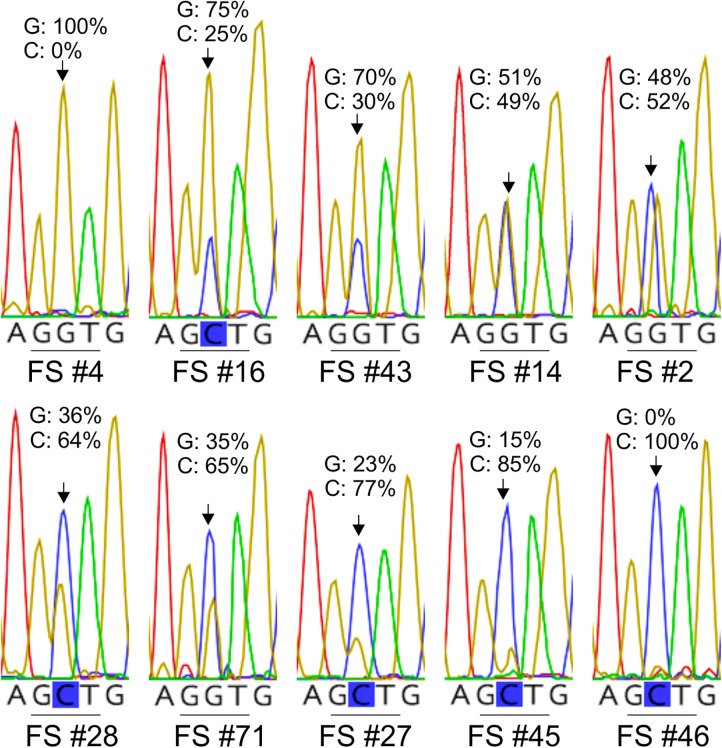
Examples of electropherograms showing different height ratios between the trace peaks for the G and C nucleotides at the nucleotide position that leads to the G143A substitution in the cytochrome b of *L. taurica* (*Lt* cytb). The electropherograms were obtained by direct sequencing of the *Lt cytb* gene fragment, following its PCR-amplification from field-derived tomato leaf samples infected with *L. taurica*. Numbering of the samples in the figure corresponds to the numbering of the samples in Supplementary Table S2. The presence of a double G/C peak indicates that the PCR product represents a mixture of wild-type (GGT: G143) and mutant (GCT: A143) cytb alleles, which may originate either from different strains of *L. taurica* present in the same leaf spot or from mitochondrial heteroplasmy in single strains of the fungus. As the height of the electrophoretic peaks at mixed base positions is relatively proportional to the quantity of the underline alleles in the heterozygous samples, the different height ratios between the G and C nucleotide peaks also indicate different ratios between the wild-type and mutated cytb allele.

Careful inspection of the electropherograms obtained from sequencing of the *Lt cytb* fragment from infected tomato leaf samples, frequently showed the presence of two overlapping electrophoretic peaks at polymorphic sites. For example, overlapping peaks for guanine (G) and cytosine (C) were frequently present at the nucleotide position that leads to the G143A substitution in the translated *Lt* cytb peptide, thus encoding both for the wild-type (GGT: G143) and mutant (GCT: A143) cytb allele (Figure 3). Moreover, the relative peak heights for the two nucleotides differed from one sample to another, indicating different ratios between the G143 and A143 alleles in the samples (Supplementary Table S2). Double electrophoretic peaks were collectively identified for all polymorphic sites in the *Lt cytb* gene fragment, except of the singletons, and in all cases the corresponding to the peaks nucleotides matched the observed for the site allelic variation. However, the distribution of the overlapping peaks was not the same across all samples, suggesting the presence of multiple cytb alleles and consequently of mitotypes within the fungal population.

A possible explanation for the presence of double electrophoretic peaks in the *cytb* sequences of nearly all of the field samples analyzed, is that most of them represented a mixture of *L. taurica* strains with different *cytb* alleles. However, although more than one strain of *L. taurica* may occur on an infected leaf, it would be surprising if almost all samples were mixtures of strains that co-existed in the same leaf spot. It is also unlikely that the observed allelic diversity originated from the amplification of the *cytb* gene of other fungal species, as this would imply that the same species were present in all samples. An alternative explanation for the presence of multiple *cytb* alleles in each sample is that of mitochondrial heteroplasmy, i.e., the coexistence of more than one type of mitochondrial DNA (mtDNA) in a single cell (Barr et al., 2005; Woloszynska, 2009; Wilson and Xu, 2012). To determine whether the presence of multiple *Lt cytb* alleles in the infected tomato leaf samples was caused by the presence of homoplasmic strains with different mitotypes or by a single *L. taurica* strain with mitochondrial heteroplasmy, we attempted to generate single spore isolates of the fungus either on tomato cv. Money Maker or cotyledons of *Lagenaria leucantha* cv. Minibottle, according to the conditions described by Guzman-Plazola et al. (2003) and Bardin et al. (2007). However, despite repeated attempts to propagate the fungus from single conidial spores, all efforts were unsuccessful. Therefore, an alternative approach was followed in which the *cytb* gene was amplified from individual single-celled conidia of *L. taurica* collected from infected leaves. This approach was also favored over the analysis of single spore isolates because the amplification of the *Lt cytb* gene fragment from individual conidia of the fungus provides stronger evidence for the presence or absence of mitochondrial heteroplasmy, as compared to amplification from bulk spores obtained from single spore isolates. PCR amplifications were performed using primer pair Lt-RSCBF1/Lt-Cytb.GSP-R2 and the PCR products were directly sequenced as before.

**FIGURE 4:**
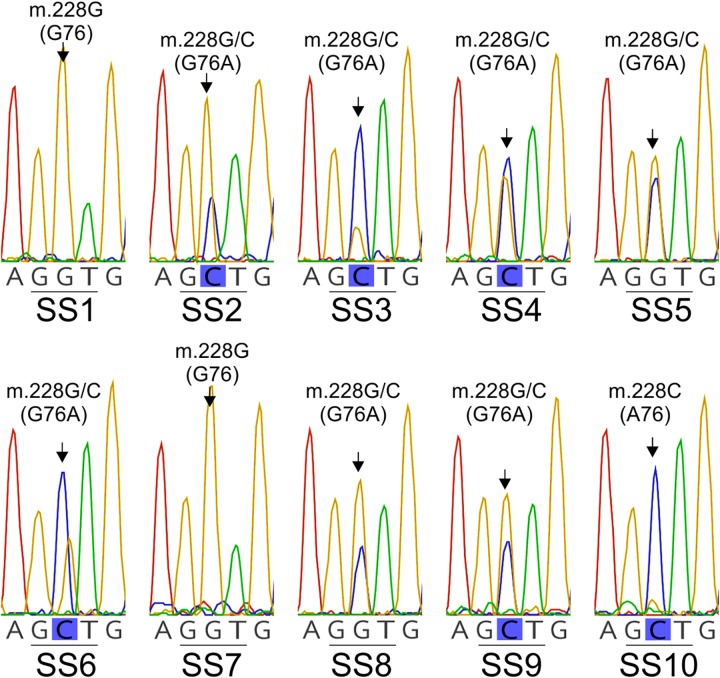
Site heteroplasmy observed in the DNA sequence electropherograms obtained by direct sequencing of the PCR amplified *Lt cytb* gene fragment from 10 single-cell conidia (SS1-to-SS10) of *L. taurica*. The partial electropherograms displayed in the figure show the presence in many cases of a double G/C peak at the nucleotide position that leads to the G143A substitution in the cytochrome b of *L. taurica* (*Lt* cytb). Note that while in some conidia the cytb is heteroplasmic (i.e., in SS2, SS3, SS4, SS5, SS6, SS8, and SS9), in others it is homoplasmic for either the wild-type (GGT: G143) (i.e., in SS1 and SS7) or mutated allele (GCT: A143) (i.e., in SS10).

Despite the low amplification rate (i.e., the Lt *cytb* gene fragment was amplified on average in only one out of ∼20 spores), the *cytb* gene was successfully amplified and sequenced from 10 individual conidia of *L. taurica* (SS1-to-SS10) (Table 2). Analysis of the obtained sequencing electropherograms showed again the presence of double peaks at previously-identified polymorphic sites, thus indicating that these are heteroplasmic mutations (Figure 4 and Supplementary Figure S3). Specifically, from the 21 SNPs identified in the *Lt cytb* gene fragment, 15 were present in a heteroplasmic state in at least one conidium, indicating high levels of heteroplasmy in this fungus (Table 2 and Supplementary Figure S3). The number of heteroplasmic sites in the *Lt cytb* gene fragment varied greatly per conidium and ranged from 0 (SS1, SS7, and SS10), to one (SS3 and SS8), two (SS9), three (SS5), four (SS4), 10 (SS6), and even 13 (SS2) sites. Notably, the most frequently observed point heteroplasmy in the chromatographic data was a double G/C peak at the nucleotide position that leads to the G143A amino acid substitution. This site was heteroplasmic in seven conidia, in contrast to other sites that were heteroplasmic in only one, two, or three conidia (Table 2 and Figure 4). Close inspection of the electropherograms also showed that the height ratio of the electrophoretic peaks for the G and C nucleotides, which is indicative of the proportion of each nucleotide in the underlying sample (Carr et al., 2009; Roy and Schreiber, 2014), varied among individual conidia (Figure 4), suggesting that the relative frequency of wild-type versus mutant *cytb* alleles differs from one conidium to the other. Moreover, while two of the conidia (SS1 and SS7) were homoplasmic for the G nucleotide, a third (SS10) was homoplasmic for the C nucleotide, indicating that in some conidia the G143A mutation is fixed. However, no conclusions could be made on allele frequency as the *Lt cytb* gene fragment was amplified from only 10 individual conidia.

Electropherograms obtained from the amplification of the *Lt cytb* gene fragment from individual single-cell conidia, also showed two new heteroplasmic mutations (i.e., m.109C>T and m.410A>G), which were absent in the *cytb* sequences obtained from the field-derived samples (Table 2, Figure 2, and Supplementary Figure S3). One of these SNPs (i.e., m.109C>T) triggers a silent substitution in the translated cytb protein (i.e., Y36Y or Y103Y with reference to the *Sc* cytb; hereafter Y103Y), but the second one (m.410A>G) results in the non-synonymous Ser-to-Gly substitution at codon 137 (S137G). Notably, the corresponding amino acid is missing in the *Sc* cytb but is present in other filamentous fungi, as seen in the amino acid alignments of their cytb proteins (Fisher et al., 2004) (Figure 2). It is plausible that these are rare alleles that were masked in *Lt cytb* sequences obtained from samples in which bulk of spores were used for DNA isolation, as their abundance relative to the dominant alleles was too low to trigger a base call. In this respect, it is suggested that with Sanger DNA sequencing the proportion of a rare nucleotide variant should be higher than 5% in order to be called as a second peak in a heteroplasmic position and be distinguished from the background noise (Carr et al., 2009; Ramos et al., 2013; Roy and Schreiber, 2014). Alternatively, these polymorphisms might represent PCR-introduced errors that were propagated during the amplification cycles.

### The G143A Substitution in the *Lt* cytb Is Wide-Spread in Field Populations of *L. taurica* in California

As the G143A mutation is most frequently found in a heteroplasmic state in the *Lt* cytb, it is plausible that resistance to Q_o_Is in this fungus is quantitative rather than qualitative and depends on the relative abundance of mitochondria carrying the mutant A143 (resistant) versus the wild-type G143 allele (sensitive) (Lesemann et al., 2006; Villani and Cox, 2014). Unfortunately, all our efforts to generate single spore isolates of *L. taurica* and subsequently examine how varying levels of heteroplasmy for the G143A mutation correlate with tolerance to Q_o_Is were unsuccessful. Thus, it is currently unknown whether the observed heteroplasmy in the *Lt* cytb leads to quantitative resistance to Q_o_Is and how different ratios of the wild-type (G143) versus the mutant (A143) cytb allele in individual *L. taurica* isolates correlate with the level of resistance. Despite this shortcoming, we assessed the frequency of occurrence of the A143 allele in field populations of *L. taurica* and quantified in each sample the ratio between the G143 and A143 alleles, by determining the height ratio between the trace peaks for the G and C nucleotides at the nucleotide position that leads to the G143A substitution (Figure 4). As electrophoretic peak height ratios at mixed base positions correlate with the DNA quantity of the underlining alleles in the heterozygous samples (Carr et al., 2009; Roy and Schreiber, 2014), determining their peak height ratios offers a simple method to estimate the proportion of each nucleotide in these positions and consequently the ratio of the two heteroplasmic components in the sample (Hunt et al., 2005; Van Leeuwen et al., 2008; Carr et al., 2009; Ramos et al., 2013; Roy and Schreiber, 2014). This approach is particularly useful when the quantity and quality of DNA is limiting, as was the case in this study.

The presence of the A143 allele was detected in 48% (50/104) of the samples analyzed (Supplementary Table S2), suggesting that resistance to Q_o_Is is developing in *L. taurica* in California. In the majority of the samples (42/50; 84%), the A143 allele was heteroplasmic with the G143 one, while its frequency varied from 14 to 85% across the samples, with an average of 40%. Thus, despite its prevalence in field populations of *L. taurica*, A143 was the minor allele in most of the heteroplasmic samples (29/42; 69%), suggesting that these populations may exhibit intermediate levels of resistance to Q_o_Is. However, eight samples in which only the A143 allele was detected were also found, indicating that the G173A mutation is fixed in some strains of *L. taurica*, which thus might exhibit higher resistance levels against Q_o_Is.

## Discussion

*Leveillula taurica* can significantly reduce tomato crop yields and, to mitigate its impact, Q_o_Is are widely used for its control in tomato fields in California and other parts of the world, thereby increasing the danger for resistance development against these fungicides (Jones and Thomson, 1987; Guzman-Plazola, 1997). To asses this risk, a fragment of the *Lt cytb* gene was cloned and analyzed for mutations that confer resistance to Q_o_Is in a large number of samples collected from fields across California. The sequencing analysis identified several polymorphisms within the *Lt cytb* gene fragment analyzed, indicating a high mutation rate. This is perhaps not surprising as mtDNA is believed to exhibit higher mutation rates compared to nuclear DNA, mainly as a result of less efficient DNA repair mechanisms and a more mutagenic intracellular environment that is created by the production of free radicals during mitochondrial respiration, such as reactive oxygen intermediates (ROIs) (Palmer et al., 2000; Gillooly et al., 2005; Lynch et al., 2006; Haag-Liautard et al., 2008). In this respect, the inhibition of mitochondrial respiration by Q_o_Is could increase mutation rates in mtDNA by triggering the accumulation of mutagenic free radicals (Bohr and Anson, 1999). However, deciphering whether the relatively large number of mutations identified in the *Lt cytb* gene fragment is a product of impaired mitochondrial activity caused by the Q_o_Is will require further work.

The analysis of the amino acid substitutions present in the *Lt* cytb peptide indicated that the notorious G143A substitution that leads to complete resistance against Q_o_Is in fungal plant pathogens (Gisi et al., 2002; Fernández-Ortuño et al., 2010), is frequently present in field populations of *L. taurica* from California, suggesting that resistance is perhaps developing in this State. However, the analysis also revealed that, like most of the allelic variation identified in the *Lt cytb*, the G143A mutation was predominately heteroplasmic and that mtDNA heteroplasmy for the wild-type and mutant cytb alleles was maintained at various levels in individual conidia as well as field samples of the fungus. The high levels of heteroplasmy observed for the G143A mutation, suggests that resistance to Q_o_Is in the fields, if present, is not yet complete but may correlate with the proportion of mitochondria in each strain carrying the mutant A143 versus the wild-type G143 cytb allele (Gisi et al., 2002; Lesemann et al., 2006). Although most fungi exhibit qualitative resistance against Q_o_Is, similar cases of quantitative resistance responses to Q_o_Is in conjunction with the heteroplasmic levels for the G143A mutation have been described in a number of plant pathogenic fungi, including the apple powdery mildew fungus *P. leucotricha* (Lesemann et al., 2006), the cucurbit powdery mildew pathogen *Podosphaera xanthii* (Vielba-Fernandez et al., 2018), the apple scab pathogen *V. inaequalis* (Villani and Cox, 2014), and the gray mold disease fungus *Botrytis cinerea* (Hashimoto et al., 2015). The high levels of heteroplasmy for the G143A mutation along with the low number of field samples in which the A143 cytb allele was fixed (8/104; 7.7%) even after a decade of use of Q_o_Is against tomato powdery mildew in California, could also imply a fitness penalty associated with the G143A mutation in *L. taurica*. If that is the case, then it is possible that the frequency of the A143 allele might increase in a cell under selection pressure from exposure to Q_o_Is, but then decrease or revert back to a homoplasmic G143 state when selection is lifted (Zheng et al., 2000; Fraaije et al., 2002). Thus, implementing Q_o_I-free periods could likely preserve the efficacy of these compounds against *L. taurica* by restoring the sensitivity levels in populations of the fungus and averting the fixation of Q_o_I-resistant strains in the fields.

Despite the prevalence of the G143A substitution within field populations of the fungus, in contrast, no substitutions were observed at residues F129L and G137R that also confer tolerance to Q_o_Is (Gisi et al., 2002). Instead, a Leu-to-Thr substitution was present at codon 130 (L130T) with reference to the *Sc* cytb, a highly conserved residue among fungal cytb protein sequences in the critical cd loop region of the protein (Fisher and Meunier, 2008) (Figure 1). The impact of this substitution on sensitivity toward Q_o_Is is currently unknown but based on its position within the Q_o_ site of cytb and its proximity to residue F129 and other residues within the cd loop region that form the enzyme’s binding pocket for strobilurins (Iwata et al., 1998; Zhang et al., 1998; Hunte et al., 2000; Fisher and Meunier, 2008), it is possible that it may affect tolerance to these compounds as well. Moreover, the biochemical properties of the large aliphatic (non-polar) amino acid Leu differ considerably from those of the smaller polar amino acid Thr, and thus exchanges between the two may lead to changes in the conformation of the enzyme’s binding pocket for Q_o_Is.

Interestingly, next to the L130T substitution, three other non-synonymous substitutions observed in the partial *Lt* cytb peptide sequence analyzed also involve exchanges between Leu and Thr, i.e., T210L and T290L, with T290 also being substituted by Phe (T290F) in some of the samples (Table 1). T210 is located within the quinone reduction (Q_i_) site (or center N) of cytb, which forms one of the two catalytic quinone-binding sites required for the Q-cycle (Figure 1) (Iwata et al., 1998; Zhang et al., 1998; Hunte et al., 2000; Fisher and Meunier, 2008). Mutations at the Q_i_ site are not expected to affect sensitivity to Q_o_ inhibitors such as strobilurins but may have an effect on resistance development toward Q_i_ inhibitors (Q_i_Is) such as cyazofamid, currently the only Q_i_I fungicide registered for use in the fields in the United States (Gentile et al., 2002; Fisher and Meunier, 2008). In a similar way, T290 is located within the transmembrane helix F1 (amino acids tracts 288–300 in the *Sc* cytb) that proceeds helix ef at the Q_o_ site (Fisher and Meunier, 2008) (Figure 2), and thus it is not expected to affect tolerance against Q_o_Is as well. Note that the corresponding L210 and L290 residues in fungal cytb sequences are highly conserved, as multiple protein alignment of the top 100 blast hits obtained using the *Lt* cytb peptide sequence as query showed that modifications in these residues were only present in three other species, i.e., two (*G. lineola* and *Gomphillus americanus*) that exhibit a Val at position L210 and one (*Juglanconis juglandina*) that exhibits a Met at position L290. Thus, it may be assumed that Leu is the expected amino acid at codons 210 and 290 of the *Lt* cytb peptide sequence, despite the fact that the majority of the samples sequenced exhibit a Thr in that position (Supplementary Table S2).

Next to the F129L, G137R, and G143A substitutions, a number of other mutations in the Q_o_ region of fungal cytb proteins have been described that affect resistance to Q_o_Is (Gisi et al., 2002; Fisher and Meunier, 2008). In particular, the L150F, I269M,L and L275F,S,T substitutions are shown to confer resistance to atovaquone in the malaria parasite *Plasmodium yoeli* (L150F and I269M) and to Myxothiazol in yeast (L275F,S,T) (Fisher and Meunier, 2008). Interestingly, L150, I269, and L275 are substituted by a Thr (T83), Met (M203), and Thr (T209), respectively, in the *Lt* cytb (Figure 2), suggesting that this species might be naturally tolerant to some Q_o_Is. L150 is located just off of the cd loop at the Q_o_ pocket, whereas I269 and L275 are located prior and at the highly conserved PEWY/ef loop, respectively (Figure 2). All three residues are also highly conserved among fungal cytb protein sequences as none other fungal species among the top 100 BLAST hits obtained using the *Lt* cytb peptide sequence as query showed modifications in these two amino acids. Apart from these three substitutions and the G143A one, none of the other mutations that are reported to affect sensitivity to Q_o_Is (i.e., A126T, F128L, G137R, M139L, I147F,V, T148I, S152A, N256Y, P266L, F278I, Y279C,S, and L282V) (Fisher and Meunier, 2008) were present in the characterized cytb fragment of *L. taurica*.

## Conclusion

In this study we report the partial cloning of the *cytb* gene from *L. taurica* and demonstrate that strains with mutations conferring resistance to Q_o_Is, such as the notorious G143A substitution that leads to complete resistance against these compounds, are already widely present across fields in California. However, the presence of this mutation in *L. taurica* is almost always associated with mitochondrial heteroplasmy, suggesting quantitative rather qualitative resistance responses to Q_o_Is. It further suggests a possible fitness penalty associated with the G143A mutation in *L. taurica*, and thus it might be possible to revert heteroplasmic isolates back to wild-type and avert the fixation of Q_o_I-resistant strains in the fields by implementing Q_o_I-free periods.

## Author Contributions

BA and IS contributed to the conceptualization. BA, EM, and IS contributed to the primary data collection and analysis and funding acquisition. SM, L-HC, AS, T-CC, LE, and IS contributed to the secondary data collection and analysis. BA and IS contributed to the methodology and project administration. BA and EM contributed to the materials. SM and IS wrote the original draft of the manuscript. L-HC, BA, EM, AS, T-CC, and LE contributed to the reviewing and editing of the manuscript.

## Conflict of Interest Statement

The authors declare that the research was conducted in the absence of any commercial or financial relationships that could be construed as a potential conflict of interest.
