# Supplementary material for: Cloning of the Cytochrome b Gene From the Tomato Powdery Mildew Fungus Leveillula taurica Reveals High Levels of Allelic Variation and Heteroplasmy for the G143A Mutation
Source: Front Microbiol. 2019 Apr 10;10:663. doi: 10.3389/fmicb.2019.00663 (PMC6467933; doi:10.3389/fmicb.2019.00663)
Supplement: Supplementary file 1 [file Data_Sheet_1.PDF]

"Cloning of the cytochrome *b* gene from the tomato powdery mildew fungus *Leveillula taurica* reveals high levels of allelic variation and heteroplasmy for the G143A mutation".

Sandra Mosquera[1†], Li-Hung Chen[1†], Brenna Aegerter[2], Eugene Miyao[3], Anthony Salvucci[1,4], Ti-Cheng Chang[1,5] and Ioannis Stergiopoulos[1,*]

Supplementary Figures and Tables

Supplementary Figure 1. Specificity of the species-specific primers designed to amplify the internal transcribed spacer (ITS) region of *Leveillula taurica* (Panel A), *Oidium lycopersici* (Panel B), and *Oidium neolycopersici* (Panel C), or the *cytb* gene from *L. taurica* (Panel D). Genomic DNA was isolated from tomato leaves infected with *L. taurica* (lanes 1-2), *O. lycopersici* (lanes 3-4), and *O. neolycopersici* (lane 5) and was used as a template in PCRs targeting the amplification of the species ITS or *cytb* sequences using the designed species-specific primers. **(A)** PCR amplification using primer pair Lt.ITS-F1/Lt.ITS-R1 designed to amplify the ITS region of *L. taurica* generated the expected 566 bp product only from samples infected with *L. taurica* (lanes 1 and 2) and not with *O. lycopersici* (lanes 3 and 4) or *O. neolycopersici* (lane 5). **(B)** PCR amplification using primer pair Ol.ITS-F2/Ol.ITS-R1 designed to amplify the ITS region of *O. lycopersici* generated the expected 406 bp product only from samples infected with *O. lycopersici* and not with *L. taurica* or *O. neolycopersici*. **(C)** PCR amplification using primer pair On.ITS-F1/On.ITS-R1 designed to amplify the ITS region of *O. neolycopersici* generated the expected 552 bp product only from samples infected with *O. neolycopersici* and not with *L. taurica* or *O. lycopersici*. **(D)** PCR amplification using primer pair RSCBF1/Lt-Cytb.GSP-R2 designed to amplify the *cytb* of *L. taurica* generated the expected 798 bp product only from samples infected with *L. taurica* and not with *O. lycopersici* or *O. neolycopersici*. PCR controls (absence of DNA from the reactions) are shown in lanes 5 and 6. Primer sequences are given in **Table S1**.

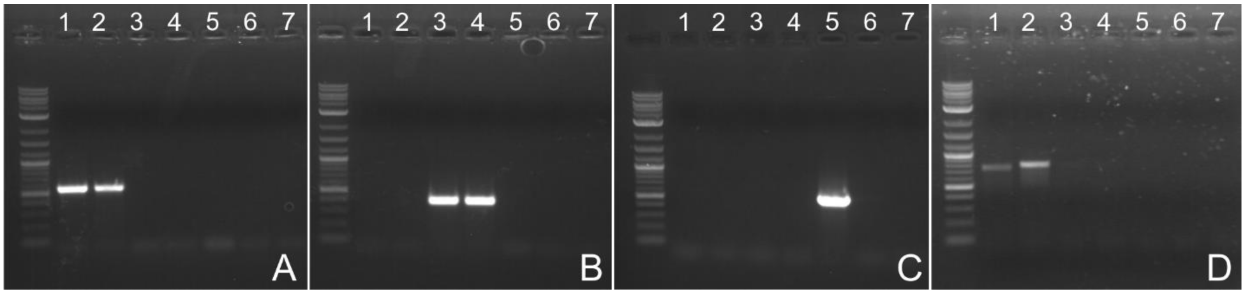

Supplementary Figure 2. The nucleotide sequence of the 837 bp *cytb* fragment obtained from *Leveillula taurica* (*Lt cytb*) by PCR using primers RSCBF1 and Cytb-MilDeg-R3, and its translated 278 amino acid product based on the yeast mitochondrial code. Positions of primers Lt-RSCBF1, Lt-RSCBR2, Lt-CytB.GSP-R2, and Cytb-MilDeg-R3 are indicated by underlined nucleotides. Single nucleotide substitutions (SNPs) identified in the *Lt cytb* amplified from field-derived samples of *L. taurica* are highlighted in different colors; red represents SNPs that result in non-synonymous substitutions in the produced cytb, whereas green and yellow SNPs that induce silent substitutions. All polymorphic nucleotide sites were found to be heteroplasmic in at least one of the field samples but only the green and red ones were heteroplasmic in *cytb* sequences amplified from single-cell conidia of *L. taurica*. Blue are nucleotides that were detected as heteroplasmic only in *cytb* sequences amplified from single-cell conidia of *L. taurica* but not from the field-derived samples.

DNA sequence:

Lt-RSCBF1 Lt-RSCBR2

TATTATGAGAGATGTAAATAATGGATGATTAATACGTTACCTACACTCAAATACAGCTTCAGCCTTTTTCTTCTTAGTTTAC
TTACACATAGGAAGAGGTTTATACTACGGGTCATACAGAGCGCCAAGAACATTAGTATGAACTATAGGTGTTGTAATATTTA
TATTAATGATGGCTACAGCTTTCTTAGGTTACGTTTTACCATACGGGCAAATGTCATTATGAGGTGCAACAGTTATTACTAA
CCTTATGAGTGCTATA**CCATGAGTAGGACAAGATATAGTT**GAATTTATTTGAGGAGGTTTCTCTGTAAATAACGCAACTTTA
AACAGATTTTTCGCATTACACTTTGTATTACCTTTTGTATTAGCTGCATTAGCATTAATGCATTTAATAGCATTACACGATA
GTGCAGGATCAGGTAATCCATTAGGTGTTTCAGGTAATTACGATAGATTACCTTTTGCTCCATACTTCATATTTAAAGATTT
AATAACTATATTCTTATTTATCGTAGTATTATCTGTTTTTGTTTTCTTTATGCCTAATGTTTTAGGTGATAGTGATAATTAT
ATTATGGCTAACCCAATGCAAACTCCACCTGCTATAGTACCTGAATGATATCTATTACCATTCTATGCTATATTAAGATCTA
TACCTAATAAATTATTAGGAGTTATATCAATGTTTGCTGCTATTTTAATTTTATTAGTTATGCCATTTACAGATTTAGGTAG
AAGTAGAGGTTTACAATTTAGACCTTTAAGTAAAATA**GCTTTCTTTATCTTTGTAGCTAA**CTTTTTAATATTAATGCAAATA
GGTGCTAGACACGTTGA

Cytb-MilDeg-R3

Lt-CytB.GSP-R2

Translated Peptide:

```
IMRDVNNGWLMRYTHSNTASAFFFLVYLHMGRGLYYGSYRAPRTLVWTMGVVMFMLMMATAFLGYVLPYGQMSLWGATVITN
TMSAMPWVGQDMVEFIWGGFSVNNATLNRFFALHFVLPFVLAALALMHLMALHDSAGSGNPLGVSGNYDRLPFAPYFMFKDL
MTMFLFIVVLSVFVFFMPNVLGDSDNYIMANPMQTPPAMVPEWYTLPFYAMLRSMPNKLLGVMSMFAAILILLVMPFTDLGR
SRGLQFRPLSKMAFFIFVANFLMLMQMGARHV
```

Translation aligned on DNA sequence (Frame 2: 5'–3'):

```
tattatgagagatgtaaataatggatgattaatacgttacctacactcaaatacagcttcagcctttttCttcttag
  I  M  R  D  V  N  N  G  W  L  M  R  Y  T  H  S  N  T  A  S  A  F  F  F  L
tttacttacacataggaagaggtttatactaCgggtcatacagagcGccAagaacattagtAtgaacTataggtgtt
V  Y  L  H  M  G  R  G  L  Y  Y  G  S  Y  R  A  P  R  T  L  V  W  T  M  G  V
gtaatatttatattaatgatggctacagctttcTtaggttacgttttaccatacggGcaaatgtcattatgagGtgc
 V  M  F  M  L  M  M  A  T  A  F  L  G  Y  V  L  P  Y  G  Q  M  S  L  W  G  A
aacagttattactaaccttatgagtgcTataccatgagtaggacaagatatagttgaatttatttgaggaggtttct
  T  V  I  T  N  T  M  S  A  M  P  W  V  G  Q  D  M  V  E  F  I  W  G  G  F
ctgtAaataacgcaactttaaacagattttCgcattacactttgtattaccttttgtattagctgcattagcAtta
S  V  N  N  A  T  L  N  R  F  F  A  L  H  F  V  L  P  F  V  L  A  A  L  A  L
```

atgcatttaatagcattacacgat**A**gtgcagg**A**tcaggtaatcca**T**taggtgtttcaggtaattacgatagattacc

M H L M A L H D **S** A **G** S G N P **L** G V S G N Y D R L P

ttttgctcc**A**tacttcatatttaaagatttaataactatattcttatttatcgtagtattatctgtttttgttttct

F A **P** Y F M F K D L M T M F L F I V V L S V F V F

ttatgcctaatgttttaggtgatagtgataa**T**ta**T**attatggctaaccc**A**atgcaaactccacctgctatagtacct

F M P N V L G D S D **N** **Y** I M A N **P** M Q T P P A M V P

gaatgatatctattaccattctatgctatattaagatctatacctaataaatta**T**t**T**gg**A**gttatatcaatgtttgc

E W Y T L P F Y A M L R S M P N K L **L** **G** V M S M F A

tgctattttaattttattagttatgccatttacagatttaggtagaagtagaggtttacaatttagacctttaagta

A I L I L L V M P F T D L G R S R G L Q F R P L S

aaatagctttctttatctttgtagctaacttttttaatattaatgcaaataggtgctagacacgttga

K M A F F I F V A N F L M L Q M G A R H V

Supplementary Figure 3. Nucleotide alignment of the DNA sequence chromatograms obtained from direct sequencing of the PCR-amplified *Lt cytb* fragment from 10 singe-cell conidia (SS1-to-SS10) of *Leveillula taurica*. Heteroplasmic sites are highlighted by a star sign. A black star indicates sites that were found to be heteroplasmic in the field samples as well, whereas a blue star indicates sites that were found to be heteroplasmic only in the conidia analyzed. Due to its very large size, the original image needs to be viewed with a picture viewing software in order to be seen in detail.

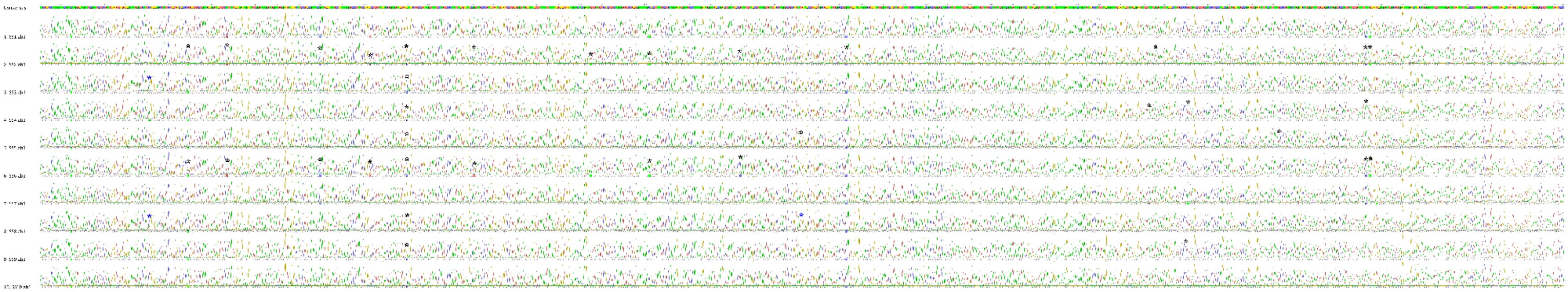

Supplemental Table 1. List of primers used in this study.

Primer	Sequence 5'–3'	Description
ITS1	TCC GTA GGT GAA CCT GCG G	Generic forward primer used for the amplification of fungal rDNA sequences
ITS4	TCC TCC GCT TAT TGA TAT GC	Generic reverse primer used for the amplification of fungal rDNA sequences
Lt.ITS-F1	CAG AGC GTG AAG ACC TCG G	Forward primer designed on the rDNA sequence of *Leveillula taurica* for the amplification of the ITS region.
Lt.ITS-R1	CCA GAA GAA GTA CAA AAG TCG CC	Reverse primer designed on the rDNA sequence of *Leveillula taurica* for the amplification of the ITS region.
Ol.ITS-F2	TGCACCGACCGGCTTC	Forward primer designed on the rDNA sequence of *Oidium lycopersici* for the amplification of the ITS region.
Ol.ITS-R1	TAG AAG GAT TGA TTG GCT AGC TGC	Reverse primer designed on the rDNA sequence of *Oidium lycopersici* for the amplification of the ITS region.
On.ITS-F1	CAG AGC GTG AGG CTC AGT C	Forward primer designed on the rDNA sequence of *Oidium neolycopersici* for the amplification of the ITS region.
On.ITS-R1	CCT GTG ATC CAT GTG ACT GGA AC	Reverse primer designed on the rDNA sequence of *Oidium neolycopersici* for the amplification of the ITS region.
RSCBF1	TAT TAT GCG TGA TGT AAA TAA TGG	Generic forward primer used for the amplification of fungal *cytb* sequences
RSCBR2	AAC AAT ATC TTG TCC AAT TCA TGG	Generic reverse primer used for the amplification of fungal *cytb* sequences
Lt-RSCBF1	TAT TAT GAG AGA TGT AAA TAA TGG	Adjusted sequence of primer RSCBF1 based on the *L. taurica c*ytb sequence
Lt-RSCBR2	AAT TAT ATC TTG TCC TAC TCA TGG	Adjusted sequence of primer RSCBR2 based on the *L. taurica c*ytb sequence

Cytb-MilDeg-F1	ATG AGA WTW TTW AAR AG	Degenerate forward primer designed based on nucleotide alignments of various fungal *cytb* genes
Cytb-MilDeg-F2	AAR AGY CAY CCH YTW TT	Degenerate forward primer designed based on nucleotide alignments of various fungal *cytb* genes
Cytb-MilDeg-F3	CCH YTW TTA ARR WTR G	Degenerate forward primer designed based on nucleotide alignments of various fungal *cytb* genes
Cytb-MilDeg-R1	TCH AYW AAA STR TTY TC	Degenerate reverse primer designed based on nucleotide alignments of various fungal *cytb* genes
Cytb-MilDeg-R2	TAC WWA WTT GWC CWA RYT C	Degenerate reverse primer designed based on nucleotide alignments of various fungal *cytb* genes
Cytb-MilDeg-R3	TCH ACR TGW YWW GCH CC	Degenerate reverse primer designed based on nucleotide alignments of various fungal *cytb* genes
Lt-Cytb.GSP-R2	TTA GCT ACA AAG ATA AAG AAA GC	Reverse primer located at the 3'-end of the *L. taurica cytb* being nested to Cytb-MilDeg-R3
Lt_cytb_5RACE_R1	CCC GTA GTA TAA ACC TCT TCC	Reverse primer used in genome walking attempts (primary PCR)
Lt_cytb_3RACE_F1	GGC TAA CCC AAT GCA AAC TCC A	Forward primer used in genome walking attempts (primary PCR)
Lt_cytb_5end_R3	CTA AAC AAA AAC CAA GTA ATG AAC C	Reverse primer used in genome walking attempts (nested PCR)
Lt_cytb_3RACE_F2	AGT ACC TGA ATG ATA TCT ATT ACC ATT CTA TGC	Forward primer used in genome walking attempts (nested PCR)
AP1	GTA ATA CGA CTC ACT ATA GGG C	Adaptor primer used with the Universal Genome Walker 2.0 kit (Clontech)
AP2:	ACTATAGGGCACGCGTGGT	Adaptor primer used with the Universal Genome Walker 2.0 kit (Clontech)

Supplemental Table 2. List of the field samples from which the cytochrome *b* of *Leveillula taurica* was amplified and analyzed in this study.

No.	Field	County	Tomato variety	Treatment	Collection date	Polymorphic sites[1] CGAATTGGTACAATATTATAA	Cytb mutations % G143A
1	Union Island, Calpack Rd.	San Joaquin	AB0311 or DR1319	Quadris Sulfur dust	7/21/2015	T...ACA.ATTC.C....CT.	G: 100% A: 0%
2	South Stockton, Mariposa Rd. & Highway 99	San Joaquin	H8504	Quadris Top Sulfur dust	7/22/2015	.ATT.C............CT.	G: 48% A: 52%
3	South Stockton, Mariposa Rd. & Highway 99	San Joaquin	H8504	Quadris Top Sulfur dust	7/22/2015	T...ACA.A.T..C.....T.	G: 100% A: 0%
4	Banta Rd, Tracy, CA.	San Joaquin	Not available	Not available	7/29/2015	T...ACA.ATTC.C....CT.	G: 100% A: 0%
5	Banta Rd, Tracy, CA.	San Joaquin	Not available	Not available	7/29/2015	T...ACA.ATTC.C....CT.	G: 100% A: 0%
6	Banta Rd, Tracy, CA.	San Joaquin	Not available	Not available	7/29/2015	T...ACA..TTC.C.......	G: 100% A: 0%
7	Banta Rd, Tracy, CA.	San Joaquin	Not available	Not available	7/29/2015	T...ACA.ATTC.C....CT	G: 100% A: 0%
8	Banta Rd, Tracy, CA.	San Joaquin	Not available	Not available	7/29/2015	T...ACA.ATTC.C....CT.	G: 100% A: 0%
9	Banta Rd, Tracy, CA.	San Joaquin	Not available	Not available	7/29/2015	T...ACA...T..C....CT.	G: 100% A: 0%
10	Banta Rd, Tracy, CA.	San Joaquin	Not available	Not available	7/29/2015	T...ACA.ATTC.C....CT.	G: 100% A: 0%
11	Union Island, Tracy Blvd. at Grant Line Canal	San Joaquin	Not available	Sulfur dust	7/24/2015	T...ACA.ATTC.C....CT.	G: 100% A: 0%
12	Union Island, Tracy Blvd. at Grant Line Canal	San Joaquin	Not available	Sulfur dust	7/24/2015	T...ACA.ATTC.C....CT.	G: 100% A: 0%

13	Union Island, Tracy Blvd. at Grant Line Canal	San Joaquin	Not available	Sulfur dust	7/24/2015	T..TA.A...T..C....CT.	G: 100% A: 0%
14	Langston Rd. 17x96 NE	Yolo	Not available	Not available	8/26/2015	T...A.......C.......	G: 51% A: 49%
15	Langston Rd. 17x96 NE	Yolo	Not available	Not available	8/26/2015	T.T..C.......C...T...	G: 68% A: 32%
16	Langston Rd. 17x96 NE	Yolo	Not available	Not available	8/26/2015	T.T.AC.......C...TC..	G: 75% A: 25%
17	Tkv	Solano	Not available	Not available	8/17/2015	T...ACA.ATTC.C....CT.	G: 100% A: 0%
18	Tkv	Solano	Not available	Not available	8/17/2015	T...A.A.ATTC.C....CT.	G: 100% A: 0%
19	Tkv	Solano	Not available	Not available	8/17/2015	T.T...A......C....CT.	G: 81% A: 19%
20	Tkv	Solano	Not available	Not available	8/17/2015	T...ACA.ATTC.C....CT.	G: 100% A: 0%
21	Tkv	Solano	Not available	Not available	8/17/2015	T...ACA.ATTC.C....CT.	G: 70% A: 30%
22	FG Field #9	Yolo	Not available	Not available	8/17/2015	T...A.A.ATTC.C....CT.	G: 100% A: 0%
23	FG Field #9	Yolo	Not available	Not available	8/17/2015	T...A.A.A.T..C....CT.	G: 100% A: 0%
24	FG Field #9	Yolo	Not available	Not available	8/17/2015	T...ACA......C....CT.	G: 100% A: 0%
25	H 25Ax98	Yolo	Not available	Not available	8/19/2015	.A.T.C.C.........CT.	G: 0% A: 100%
26	H 25Ax98	Yolo	Not available	Not available	8/19/2015	T...A.A.ATTC.C....CT.	G: 59% A: 41%
27	H 25Ax98	Yolo	Not available	Not available	8/19/2015	T....CAC.TTC.C....C..	G: 23% A: 77%
28	H 25Ax98	Yolo	Not available	Not available	8/19/2015	T...ACAC..T..C....CT.	G: 36% A: 64%
29	H 25Ax98	Yolo	Not available	Not available	8/19/2015	T.T.ACA.ATTC.C.....T.	G: 59% A: 41%
30	H 25Ax98	Yolo	Not available	Not available	8/19/2015	T...A.ACA.TC.C....CT.	G: 40% A: 60%
31	H 25Ax98	Yolo	Not available	Not available	8/19/2015	T.T...AC.....C....C..	G: 31% A: 69%
32	Armstrong	Solano	Not available	Not available	8/19/2015	T...A.A.A....C.....T.	G: 72% A: 28%
33	Armstrong	Solano	Not available	Not available	8/19/2015	T...ACA..TTC.C....CT.	G: 100% A: 0%

34	Armstrong	Solano	Not available	Not available	8/19/2015	T...A.A.ATTC......CT.	G: 100% A: 0%
35	Armstrong	Solano	Not available	Not available	8/19/2015	T.T.........C.......	G: 100% A: 0%
36	Armstrong	Solano	Not available	Not available	8/19/2015	T...A.A.ATTC.C....CT.	G: 100% A: 0%
37	Armstrong	Solano	Not available	Not available	9/9/2015	T...ACA...T..C....CT.	G: 100% A: 0%
38	Armstrong	Solano	Not available	Not available	9/9/2015	T...A.A.ATTC.C....CT.	G: 100% A: 0%
39	Armstrong	Solano	Not available	Not available	9/9/2015	T.T.ACA.ATTC.C....CT.	G: 100% A: 0%
40	Armstrong	Solano	Not available	Not available	9/9/2015	T.T...A..T.C.C.......	G: 100% A: 0%
41	Armstrong	Solano	Not available	Not available	10/28/2015	T...A.A.ATTC.C....CT.	G: 100% A: 0%
42	Coney Island	Contra Costa	Not available	Quadris Top<br>Sulfur<br>Quintec	8/19/2015	T.T.A.A..TT..C.......	G: 76% A: 24%
43	Farmington Rd at Jack Tone Rd	San Joaquin	N6407	Quadris Top<br>Cabrio<br>Quadris Top	8/20/2015	T.T....C.....C.......	G: 50% A: 50%
44	Farmington Rd at Jack Tone Rd	San Joaquin	N6407	Quadris Top<br>Cabrio<br>Quadris Top	8/20/2015	T.T..C.......C.......	G: 100% A: 0%
45	98 x 29 NW	Yolo	Not available	Not available	8/31/2015	T..TA.ACATTC.C....CT.	G: 15% A: 85%
46	98 x 29 NW	Yolo	Not available	Not available	8/31/2015	T.T....C.....C.......	G: 0% A: 100%
47	98 x 29 NW	Yolo	Not available	Not available	8/31/2015	T.T....C.....C.......	G: 25% A: 75%
48	Rd 11 x I-5	Yolo	Not available	Not available	8/31/2015	T...ACAC.TTC.C....CT.	G: 50% A: 50%
49	Rd 11 x I-5	Yolo	Not available	Not available	8/31/2015	T.T....C.....C.......	G: 20% A: 80%
50	Rd 11 x I-5	Yolo	Not available	Not available	8/31/2015	T...A.AC.T...C.....T.	G: 42% A: 58%
51	Rd 11 x I-5	Yolo	Not available	Not available	8/31/2015	T.T....C.....C.......	G: 0% A: 100%
52	Rd 11 x I-5	Yolo	Not available	Not available	8/31/2015	T...A.A.ATTC.C....CT.	G: 76% A: 24%
53	Rd 11 x I-5	Yolo	Not available	Not available	8/31/2015	TA.T.CAC.T.C......CT.	G: 0% A: 100%

54	YE-20186	Yolo	Not available	Not available	8/31/2015	T.T..........C....C..	G: 77%	A: 23%
55	YE-20186	Yolo	Not available	Not available	8/31/2015	T....C.......C....C..	G: 81%	A: 19%
56	YE-20186	Yolo	Not available	Not available	8/31/2015	T.T.ACA.ATTC.C....CT.	G: 100%	A: 0%
57	YE-20186	Yolo	Not available	Not available	8/31/2015	T...A.A.A.T..C....CT.	G: 84%	A: 16%
58	YE-20186	Yolo	Not available	Not available	8/31/2015	T.T..........C.......	G: 100%	A: 0%
59	YE-20186	Yolo	Not available	Not available	8/31/2015	T.T..........C.......	G: 71%	A: 29%
60	YE-20186	Yolo	Not available	Not available	8/31/2015	T...A.A.ATTC.C....CT.	G: 100%	A: 0%
61	Willow	Yolo	Not available	Not available	9/1/2015	T...ACA.ATTC.C....CT.	G: 100%	A: 0%
62	Willow	Yolo	Not available	Not available	9/1/2015	T.T..........C....C..	G: 80%	A: 20%
63	Willow	Yolo	Not available	Not available	9/1/2015	T...A.A...T..C....CT.	G: 100%	A: 0%
64	Willow	Yolo	Not available	Not available	9/1/2015	T...A........C.......	G: 65%	A: 35%
65	Willow	Yolo	Not available	Not available	9/1/2015	T.T...A..T.C.C....CT.	G: 76%	A: 24%
66	Willow	Yolo	Not available	Not available	9/1/2015	T.T.A.....T..C.......	G: 86%	A: 14%
67	M 104 x 29	Yolo	Not available	Not available	9/1/2015	T............C.......	G: 68%	A: 32%
68	M 104 x 29	Yolo	Not available	Not available	9/1/2015	T...A.AC..T..C....CT.	G: 0%	A: 100%
69	M 104 x 29	Yolo	Not available	Not available	9/1/2015	T...ACA.ATTC.C....CT.	G: 100%	A: 0%
70	M 104 x 29	Yolo	Not available	Not available	9/1/2015	T...A.ACATTC.C....CT.	G: 35%	A: 65%
71	M 104 x 29	Yolo	Not available	Not available	9/1/2015	T...ACA.ATTC.C....CT.	G: 61%	A: 39%
72	NW Knights Landing W Highway 45	Yolo	Not available	Not available	9/1/2015	T....CAC..T..C....C..	G: 41%	A: 59%
73	NW Knights Landing W Highway 45	Yolo	Not available	Not available	9/1/2015	T.T..........C.......	G: 100%	A: 0%
74	NW Knights Landing W Highway 45	Yolo	Not available	Not available	9/14/2015	.ATT.C............CT.	G: 60%	A: 40%
75	NW Knights Landing W Highway 45	Yolo	Not available	Not available	9/14/2015	T.TT.C.......C....CT.	G: 77%	A: 23%

76	NW Knights Landing W Highway 45	Yolo	Not available	Not available	9/14/2015	T.T..........C.......	G: 100%	A: 0%
77	H#72 N	Yolo	Not available	Not available	9/14/2015	T...A.A...T..C..C.CT.	G: 100%	A: 0%
78	Dixon S West T	Solano	Not available	Not available	9/22/2015	T.T...A......C....CT.	G: 80%	A: 20%
79	Dixon S West T	Solano	Not available	Not available	9/22/2015	T...ACA.ATTC.C....CT.	G: 100%	A: 0%
80	Fresno County - Untreated	Fresno	Not available	Untreated	9/23/2015	T...ACA.ATTC.C....CT.	G: 64%	A: 36%
81	Fresno County - Untreated	Fresno	Not available	Untreated	9/23/2015	T.T..........C....CT.	G: 100%	A: 0%
82	Fresno County - Untreated	Fresno	Not available	Untreated	9/23/2015	T...A...AT...C.....T.	G: 69%	A: 31%
83	Fresno County - Treated 6	Fresno	Not available	Not available	9/23/2015	T.T....C.....C.......	G: 0%	A: 100%
84	Fresno County - Treated 6	Fresno	Not available	Not available	9/23/2015	T.T..........C.......	G: 59%	A: 41%
85	Fresno County - Treated 6	Fresno	Not available	Not available	9/23/2015	T.T....C.....C.......	G: 0%	A: 100%
86	Fresno County - Treated NE	Fresno	Not available	Not available	9/23/2015	T.T..CA..TTC.C....CT.	G: 84%	A: 16%
87	Fresno County - Treated NE	Fresno	Not available	Not available	9/23/2015	T...A...ATTC.C....CT.	G: 84%	A: 16%
88	Fresno County - Treated NE	Fresno	Not available	Not available	9/23/2015	T.T..........C....C..	G: 58%	A: 42%
89	Fresno County - Treated NE	Fresno	Not available	Not available	9/23/2015	T...ACA...T.GCT...CT.	G: 100%	A: 0%

90	Clifton Court Rd. near Bonetti Rd.	San Joaquin	UG19406	Flutriafol	10/5/2015	`T...ACA.ATTC.C....CT.`	G: 100% A: 0%
91	Clifton Court Rd. near Bonetti Rd.	San Joaquin	UG19406	Flutriafol	10/5/2015	`T...A.A.A....C....CT.`	G: 100% A: 0%
92	Clifton Court Rd. near Bonetti Rd.	San Joaquin	UG19406	Flutriafol	10/5/2015	`T.T..........C.......`	G: 100% A: 0%
93	Clifton Court Rd. near Bonetti Rd.	San Joaquin	UG19406	Flutriafol	10/5/2015	`T.T....C.....C.......`	G: 0% A: 100%
94	Clifton Court Rd. near Bonetti Rd.	San Joaquin	UG19406	Flutriafol	10/5/2015	`T...ACA.ATTC.C....CT.`	G: 100% A: 0%
95	Clifton Court Rd. near Bonetti Rd.	San Joaquin	UG19406	Untreated	10/5/2015	`T...ACA.ATTC.C....CT.`	G: 100% A: 0%
96	Clifton Court Rd. near Bonetti Rd.	San Joaquin	UG19406	Untreated	10/5/2015	`T...ACA.ATTC.C....CT.`	G: 100% A: 0%
97	Clifton Court Rd. near Bonetti Rd.	San Joaquin	UG19406	Untreated	10/5/2015	`T...A.A..T.C.C....CT.`	G: 100% A: 0%
98	Clifton Court Rd. near Bonetti Rd.	San Joaquin	UG19406	Untreated	10/5/2015	`T...ACA.ATTC.C....CT.`	G: 100% A: 0%
99	BA field	San Joaquin	UG19406	Untreated	08/12/2015	`.....C.......C.C.TC.T`	G: 100% A: 0%
100	BA field	San Joaquin	UG19406	Rally	08/12/2015	`.....C.......C.C.TC.T`	G: 100% A: 0%
101	BA field	San Joaquin	UG19406	Quadris	08/12/2015	`.....C.......C.C.TC.T`	G: 100% A: 0%
102	BA field	San Joaquin	UG19406	Cabrio	08/12/2015	`.....C.......C.C.TC.T`	G: 100% A: 0%
103	BA field	San Joaquin	UG19406	Quadris Top	08/18/2015	`.....C.......C.C.TC..`	G: 100% A: 0%
104	BA field	San Joaquin	UG19406	Rally	08/12/2015	`.....C.......C.C.TC.T`	G: 100% A: 0%

[1] Nucleotides correspond to the 21 polymorphic sites found in the 704 bp *cytb* fragment of *L. taurica* analyzed and arranged in the order that they appear in the sequence. Shown in the ledger is the "wild-type" sequence of *Lt cytb* and for each sample the observed nucleotide variation is indicated. Dots signify matching nucleotides with the wild-type. Note that heteroplasmy is not considered in the polymorphic data.
